# The Role and Associated Factors of Liquid-Based Cytology of Bronchoalveolar Lavage Fluid in Lung Cancer Diagnosis: A Prospective Study

**DOI:** 10.7759/cureus.48483

**Published:** 2023-11-08

**Authors:** Khoa Nguyen-Dang, Hanh-Duyen Bui-Thi, Ngoc Duong-Minh, Thong Pham-Quang, Lam Nguyen-Ho, Dung Lam-Quoc, Thong Dang-Vu, Nguyen Tran-Ngoc, Phung Nguyen-Thi, Vu Le-Thuong

**Affiliations:** 1 Department of Internal Medicine, Faculty of Medicine, University of Medicine and Pharmacy at Ho Chi Minh City, Ho Chi Minh, VNM; 2 Department of Pulmonary Medicine, Cho Ray Hospital, Ho Chi Minh, VNM; 3 Department of Intensive Care, University Medical Center Ho Chi Minh City, University of Medicine and Pharmacy at Ho Chi Minh City, Ho Chi Minh, VNM; 4 Department of Pathology, Cho Ray Hospital, Ho Chi Minh, VNM; 5 Department of Tuberculosis and Pulmonary Disease, University of Medicine and Pharmacy at Ho Chi Minh City, Ho Chi Minh, VNM; 6 Department of Respiratory Medicine, University of Medicine and Pharmacy at Ho Chi Minh City, Ho Chi Minh, VNM

**Keywords:** flexible bronchoscopy, lung cancer, bronchoalveolar lavage fluid, conventional smear, liquid-based cytology

## Abstract

Background

Liquid-based cytology (LBC) has shown advantages over conventional smears (CS), but previous applications in bronchoalveolar lavage (BAL) fluid have produced inconsistent results. This study compared LBC and CS for diagnosing lung cancer using BAL fluid.

Methodology

A prospective study was conducted on 92 patients suspected of having lung cancer. All patients underwent bronchoscopy and had a final diagnosis confirmed by histopathology of lesions tissue through biopsy. The study aimed to assess the sensitivity, specificity, positive predictive value (PPV), and negative predictive value (NPV) of the two cytological methods, in a pair-wise fashion. In addition, the study evaluated the correlation of factors, such as the volume of fluid used in LBC and bronchoscopy lesion morphology, with the sensitivity of LBC.

Results

The study involved 78 participants who were diagnosed with lung cancer. The sensitivity, specificity, PPV, and NPV of LBC were 25.7%, 100%, 100%, and 19.4%, respectively, whereas those of CS were 15.4%, 85.7%, 85.7%, and 15.4%, respectively. Although the sensitivity of LBC was higher than that of CS, the difference was not statistically significant (p=0.096, McNemar test). Furthermore, the median fluid volume performed during LBC in patients with positive results was significantly higher than in those with negative results (p=0.001, Mann-Whitney U test).

Conclusions

The application of LBC to BAL fluid has demonstrated similar and potentially superior diagnostic accuracy compared to CS in detecting lung cancer. It is recommended that further investigation be undertaken to examine the relationship between the volume of fluid utilized during the LBC process and its diagnostic accuracy to enhance its sensitivity.

## Introduction

Lung cancer is the primary cause of cancer-related deaths in numerous nations worldwide [1]. Patients with early-stage disease are often asymptomatic, and diagnosis is frequently incidental via chest imaging or lung cancer screening programs in high-risk individuals. Consequently, about 70% of lung cancers are in the progressive stage when making the diagnosis [2]. Nearly 50% of advanced cases involve the central airways, causing flexible bronchoscopy to play a crucial role in diagnosing [3]. The British Thoracic Society recommended bronchoscopic biopsy combined with bronchial brush or bronchoalveolar lavage (BAL) fluid cytology to increase the sensitivity of diagnosing lung cancer [4]. 

The conventional smear (CS) technique has been routinely applied to BAL fluid samples to assist in diagnosing lung cancer. However, this method has revealed many drawbacks, such as taking a long time to prepare the sample, the smears sometimes being too thick, and regions of overlapping cells. Moreover, inflammatory cells, blood cells, and air-drying artifacts also limited morphological observations of the nucleus and essential cells [5]. These technical challenges are thought to lead to suboptimal sensitivity of the CS method.

In recent years, liquid-based cytology (LBC) technique has been developed. It provides some significant advantages over CS, such as avoiding contamination by peripheral blood through lysis of red blood cells, inflammatory exudates, mucus, air-drying artifacts, and better cell shape preservation [6,7]. In the diagnosis of lung cancer, LBC has been applied to various types of specimens, such as sputum [7], BAL fluid [8-12], bronchial brushing [11], or transbronchial needle aspiration [13]. These studies have demonstrated that LBC has a higher sensitivity and specificity than CS, but several authors have reported contradictory results. Therefore, we conducted this study to evaluate the diagnostic value of LBC of BAL fluid in diagnosing lung cancer and compared it with CS. In addition, the study aimed to assess the factors that influence the sensitivity of LBC in detecting cells with suspected malignancy.

## Materials and methods

A prospective study was conducted from October 2021 to May 2022 at the Department of Pulmonary Medicine of Cho Ray Hospital (Ho Chi Minh City, Vietnam). The study enrolled 95 patients admitted to the hospital with suspected lung cancer with a definitive diagnosis confirmed by histopathology. Patients were identified as having suspected lung cancer based on lung nodules or masses, unexplained pleural effusion, consolidation or atelectasis of unknown origin, interstitial lung lesions suggestive of malignancy, and mediastinal lymph node with suspected malignancy (short-axis diameter larger than 1 cm) on chest computed tomography. Diagnostic flexible bronchoscopy was performed in all cases. Lesions were considered central if bronchoscopy revealed abnormal appearances and peripheral if otherwise. The endobronchial appearances on bronchoscopy were categorized according to Buccheri [14] into tumor, necrosis, inflammatory, compression, and non-specific types.

Inclusion criteria

The study recruited patients 18 years or older with suspected lung cancer based on chest computed tomography features (described above). Definite diagnosis criteria were established: i) Lung cancer diagnosis was confirmed by histopathologic examination of tissue-based biopsy. ii) Pulmonary tuberculosis was confirmed by granulomatous inflammatory tissue histopathology of lung or bronchial tissue, positive response to anti-tuberculosis therapy, and lesion resolution within three months on chest imaging. iii) Pneumonia was confirmed by inflammatory or necrotic tissue histopathology, response to antibiotics treatment, and lesion resolution within two weeks on chest imaging. iv) Alveolar microlithiasis, granulomatous disease, and acute eosinophilic pneumonia were diagnosed based on consistent histopathology results. Immunohistochemistry was performed on lung cancer cases and classified according to the World Health Organization criteria [15].

Exclusion criteria

We excluded patients with the following characteristics: i) having contraindication to flexible bronchoscopy; ii) known lung cancer prior to admission; iii) currently experiencing cancer treatment with chemotherapy, radiation therapy, or targeted therapy; iv) diagnosing metastatic cancer to the lungs.

Flexible bronchoscopy procedure

The Olympus CV-170 bronchoscope system and Olympus BF-1TQ170 bronchoscope (Olympus, Japan) were used in this study. Before the procedure, the patients received 0.25 mg of subcutaneous atropine and anesthesia for the pharynx with 2% lidocaine. The lung lobe intended for bronchoalveolar lavage was determined by the suspected malignancy appearance observed during bronchoscopy. If no abnormality was seen, the washing zone was directed by computed chest tomography. A solution of 50 mL of 0.9% NaCl was gradually injected into the bronchial lumen, and the bronchoscope was held in place while gently aspirating to collect the fluid in a sterile container. The lavage was performed up to three times based on patient tolerance, and if SpO_2_ decreased more than 4%, the procedure was terminated. A bronchial mucosa biopsy was performed if a suspected malignancy lesion was observed; otherwise, a transbronchial lung biopsy was considered. BAL fluid was transmitted for performing two cytology methods within an hour. Patients without a definite diagnosis after diagnostic bronchoscopy underwent other biopsy approaches, including ultrasound-guided percutaneous lung biopsy, percutaneous computed tomography-guided lung biopsy, pleural biopsy, endobronchial ultrasound-guided transbronchial needle aspiration, or endobronchial ultrasound-guided transbronchial lung biopsy.

Conventional smear method

The BAL fluid sample underwent centrifugation in 2000-3000 revolutions per minute for 10-15 minutes (447-1006 x G). The resulting upper layer of clear fluid was removed while retaining the residue at the bottom of the centrifuge tube. The residue was then smeared onto a slide and allowed to dry. Subsequently, the slide was stained with Wright and Giemsa for three to five minutes.

Liquid-based cytology method

In the liquid-based cytology (LBC) method, 30 mL of CytoLyt solution (Hologic, Marlborough, Massachusetts, USA) was added to the BAL fluid, followed by stirring on a mechanical mixer for five minutes and subsequent centrifugation at 1200 revolutions per minute for five minutes. After centrifugation, the supernatant was withdrawn from the top, while the components at the bottom were preserved. The cell mixture was examined for quality. If it appeared white, pale pink, or not visible, it was stabilized with a PreservCyt solution (Hologic, Marlborough, Massachusetts, USA) for 15 minutes and processed using the ThinPrep 2000 Processor machine (Hologic Corp., USA). In case the mixture seemed red or brown (i.e., blood is present) or mucilaginous, the specimen preparation steps were repeated. Finally, the slide was stained with Papanicolaou's (Pap) stain.

Report of cytology results

The results of CS were evaluated by a single laboratory technician entirely and blinded with LBC and histopathology results. In addition, the same pathologist provided the report for all LBCs without access to any other results (CS or histopathology results). The laboratory technician and pathologist possess over five years of experience identifying respiratory malignancies.

**Figure 1:**
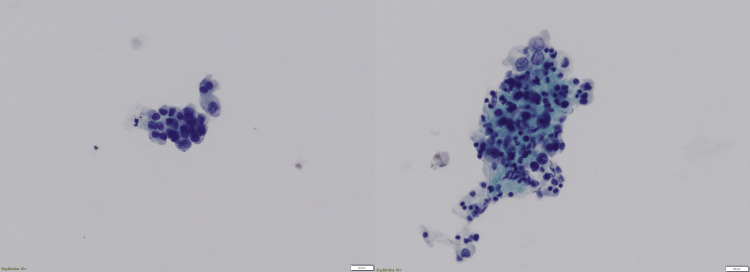
Cells with irregular nuclei were observed based on the liquid-based cytology results (Pap stain, 20X). The cells appeared crowded and overlapping. The patient was diagnosed with adenocarcinoma based on a histopathological examination of biopsy tissue samples.

**Figure 2:**
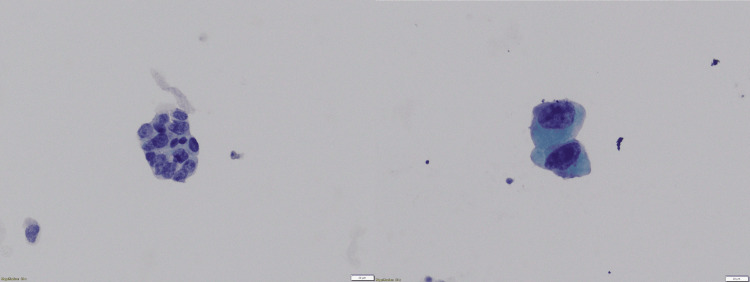
The liquid-based cytology results (Pap stain, 20X) revealed irregular and stacked cell nuclei, with many cells exhibiting coarse chromatin and prominent nucleoli. The patient was diagnosed with adenocarcinoma via histopathological examination of biopsy tissue samples.

The cytological findings were categorized into two groups: i) positive: detection of atypical cells indicative of malignancy, as illustrated in Figure 1 and Figure 2, and ii) negative: no abnormal cells were detected.

Statistical analysis

The statistical analysis was performed using Stata Statistical Software (release 14, StataCorp., 2015, College Station, TX: StataCorp LLC). Normally distributed quantitative variables are expressed as mean ± standard deviation (SD), while non-normally distributed quantitative variables are presented as medians. Qualitative variables are expressed as percentages. The sensitivity, specificity, positive predictive value (PPV), and negative predictive value (NPV) of LBC and CS were calculated. Statistical comparisons between two independent groups were conducted using Student's t-test. Mann-Whitney U test was used to compare medians between two independent groups, and McNemar test was used to compare paired data proportions in the same population. A p-value less than 0.05 was considered statistically significant.

Medical ethics

The present study obtained approval from the Ethical Review Committee, University of Medicine and Pharmacy at Ho Chi Minh City (No. 519/HĐĐĐ-ĐHYD). All patients participating in the study signed informed consent.

## Results

Patient characteristics

Ninety-five patients with suspected lung cancer participated in the study, all with at least one histopathological result to determine the etiology. Three patients diagnosed with lung metastatic cancer were excluded from the study. The study population comprised 78 (84.8%) lung cancer cases and 14 (15.2%) non-lung cancer cases (as shown in Table 1). Lung cancer patients had a mean age of 60.9 ± 12.9 years, with males comprising 60.3%. The time to symptom onset ranged from 0 to 365 days, with a median of 30 days. Two cases (2.6%) were asymptomatic and were detected through screening programs. Cough and weight loss were the most common symptoms in about 70% of cases.

**Table 1:** Histopathology of the study population (n = 92).

	Histopathology	Number of patients (%, n)
Lung cancer (N=78)	Adenocarcinoma	76.9% (60/78)
	Squamous cell carcinoma	12.8% (10/78)
	Adenosquamous carcinoma	2.6% (2/78)
	Small cell carcinoma	2.6% (2/78)
	Carcinosarcoma	1.3% (1/78)
	Neuroendocrine tumors	1.3% (1/78)
	Spindle cell carcinoma	1.3% (1/78)
	Large cell carcinoma	1.3% (1/78)
Non-lung cancer (N=14)	Pneumonia	42.9% (6/14)
	Tuberculosis	28.6% (4/14)
	Granulomatous disease	7.1% (1/14)
	Alveolar microlithiasis	7.1% (1/14)
	Lung abscess	7.1% (1/14)
	Acute eosinophilic pneumonia	7.1% (1/14)

Value of cytological methods

All cases of positive CS and LBC results had a definitive diagnosis of adenocarcinoma. The incidence of adenocarcinomas with positive LBC results was statistically significantly higher than other histopathological categories (0/18, p=0.004, Fisher's exact test). LBC has higher sensitivity, specificity, and PPV than CS (as shown in Table 2). CS had two false positives, which included pulmonary tuberculosis and pneumonia. LBC has higher sensitivity than CS, although not statistically significant (p=0.096, McNemar test). Combining both cytology methods showed a higher sensitivity than utilizing CS alone, and the difference was statistically significant (p=0.0002, McNemar test).

**Table 2:** Sensitivity, specificity, positive predictive value, and negative predictive value of the two cytological methods. CS: conventional smear, LBC: liquid-based cytology, PPV: positive predictive value, NPV: negative predictive value, ^a^McNemar test

Value	CS, N (%)	LBC, N (%)	p^a^	LBC + CS, N (%)
Sensitivity (%)	12/78 (15.4%)	20/78 (25.7%)	p=0.096	25/78 (32.1%)
Specificity (%)	12/14 (85.7%)	14/14 (100%)		
PPV (%)	12/14 (85.7%)	20/20 (100%)		
NPV (%)	12/78 (15.4%)	14/72 (19.4%)		

A total of 67 endoscopic biopsies, including 50 bronchial mucosal biopsies and 17 transbronchial lung biopsies, were performed in the study. Of these, 45 cases (39 bronchial mucosal biopsies and six transbronchial lung biopsies) were positive for cancer. Combined LBC and biopsy did not show a statistically significant increase in sensitivity (46/67 compared with 45/67 cases, p=1.00, Fisher’s exact test). In the 78 lung cancer cases, the median volume of fluid used for LBC was 5 mL, with an interquartile range of 5-10 mL. The fluid volume in the positive LBC group was significantly higher than in the negative group (median 10 mL vs. 6 mL, p=0.001, Mann-Whitney U test). Infiltrative lesions had the highest positive rate for LBC (43.7%), but there was no statistically significant difference between bronchoscopy lesion morphology and LBC results (p=0.368, Fisher’s exact test) (as shown in Table 3).

**Table 3:** Positive rate of liquid-based cytology according to lesion morphology detected through bronchoscopy. LBC: liquid-based cytology, ^a^Fisher’s exact test

Morphology	Number of patients (N)	Positive rate of LBC (n, %)	p^a^
Tumorous	18	3/18 (16.7%)	p = 0.368
Necrosis	2	0/2 (0.0%)	
Infiltrate	23	10/23 (43.7%)	
Compression	8	1/8 (12.5%)	
Non-specific	5	1/5 (20.0%)	
Normal	22	5/22 (22.7%)	

## Discussion

Several studies have compared different cytological techniques to diagnose lung cancer. Han et al. [10] applied LBC in four groups using various sampling procedures, obtaining a sensitivity of 87.1%, specificity of 60.6%, PPV of 92%, and NPV of 47.6% in the BAL fluid group using LBC. Cao et al. [11] found that LBC BAL fluid had a sensitivity of 34.9%, specificity of 100%, PPV of 100%, and NPV of 39.4%. The sensitivity of LBC declined to 12.5% in instances of peripheral lung cancer, although it increased to 69.3% compared to visible lesions [11]. Meanwhile, Yang et al. [9] compared LBC and CS BAL fluid in lung cancer diagnosis and found that LBC had a significantly higher diagnostic sensitivity (35.9% vs. 11.8%). Thakur et al. [12] also demonstrated that LBC has a higher sensitivity than CS (55.8% vs. 50.7%), with both techniques having a specificity of 100%. Kathiresan et al. [8] found that LBC had a higher sensitivity than CS (54% vs. 38%), and the BAL fluid volume did not impact the sensitivity of LBC. However, Nalwa et al. [13] reached an inconsistent conclusion, with comparable diagnostic roles observed for LBC and CS of BAL fluid (57.8% vs. 68.4%, respectively) with the same specificity of 100%, despite LBC preserving the cell morphology better.

Our study revealed that the sensitivity of LBC BAL fluid was lower than those reported in previous studies, including Cao et al. (34.9%) [11], Yang et al. (35.8%) [9], Kathiresan et al. (54%) [8], Thakur et al. (55.8%) [12], Nalwa et al. (76.7%) [13], and Han et al. (87.1%) [10]. To explain this dissimilarity, we have the following hypotheses. First, in our study, BAL fluid was obtained before endoscopic biopsy, as endoscopic biopsy involves a risk of bleeding, bronchospasm, and respiratory failure. As a result, there is a potential hazard that patients may not tolerate the irrigation of 0.9% normal saline into the airways after a successful biopsy. Pirozynski [16] and Yang et al. [9] collected BAL fluid after biopsies, expecting more malignant cells to be released from the airways. Pirozynski [16] reported a CS sensitivity of 64.8%, which was higher than that of our study (16.1%), and Yang et al. [9] reported a higher LBC sensitivity of 35.9% compared to our study (24.7%). Second, Medha et al. [17] postulated that tumors in the lung parenchyma or interstitium may not shed malignant cells into the airways, unlike tumors in the alveoli or the bronchial lumen. Consequently, the cytological findings are contingent upon the histopathological characteristics of the study population. Third, Nalwa et al. [13] utilized immunohistochemistry on LBC specimens, with the possibility that the author reports a sensitivity of LBC up to 76.7%. Finally, to our knowledge, comparative studies on BAL fluid specimens using ThinPrep systems and Sure Path systems are yet to be available.

Bronchoscopy for obtaining BAL fluid offers several advantages, such as minimal invasiveness, diagnostic utility, and lower incidence of complications, including pneumothorax and bleeding. Notably, BAL fluid provides details about cells located in the peripheral airways. LBC is a newer method that produces a thin cell layer and has received FDA approval for both gynecological (typically cervical) and non-gynecological samples [5]. LBC offers various benefits that augment the diagnostic potential of cytology. These benefits include the ease of standardization of sample preparation, reducing the likelihood of partial specimen loss and false negative results, and the use of fully or partially automated sample handling with the Thinprep or SurePath systems. LBC also allows for the immediate preservation of cells and avoidance of air-drying artifacts, even the distribution of product cells, and a smaller and more focused viewing area of 20 mm with Thinprep and 13 mm with SurePath, enabling a quicker and more efficient analysis [5,6].

In this study, two patients had false-positive results with CS due to underlying inflammatory and infectious diseases, such as pneumonia and pulmonary tuberculosis. Similar cases have been reported by Ahmad et al. [18], Rao et al. [19], and Binesh et al. [20]. Cytological methods may yield false-positive results in cell proliferation, metaplasia, or degradation in lung and bronchial cells, frequently seen in inflammatory and infectious disorders [10]. For instance, reactive type II pneumocytes, observed in acute respiratory distress syndrome, may be mistaken for adenocarcinoma, leading to false-positive results [21]. Other illnesses, such as pulmonary infarction, fungal infections, and granulomatous disease, may also complicate the interpretation of cytological samples by showing atypical reactive squamous cells or keratolytic squamous cell carcinoma [22]. Underlying lesions, such as infections or granular cell tumors, can also produce a prominent reactive squamous proliferation of the mucosa (or pseudoepitheliomatous hyperplasia) that can lead to a misdiagnosis of squamous cell carcinoma [23].

Five studies compared the diagnostic value of the two cytological techniques. Nalwa et al.'s study was the only one to show higher sensitivity of CS than LBC [13]. Yang et al. [9], Kathiresan et al. [8], and Thakur et al. [12] all demonstrated higher sensitivity of LBC compared with CS. Except for Thakur et al. [12], the remaining authors found this difference statistically significant. Although our study suggests a higher sensitivity of LBC than CS, this difference is not statistically significant, possibly due to a small sample size.

Regarding the amount of BAL fluid utilized in LBC, our research demonstrated a statistically significant difference between the positive and negative groups. The former had a higher median volume of 10 mL compared to 6 mL in the latter. This could be attributed to the greater probability of capturing malignant cells in the airways with a larger fluid sample, resulting in increased sensitivity. However, Kathiresan et al. [8] reported a mean volume of 7 mL for both positive and negative groups, indicating no correlation between fluid volume and sensitivity. Further studies are necessary to confirm this hypothesis.

Furthermore, infiltrative lesions had the highest positive rate for LBC, with 10 out of 23 cases (43.5%) showing positive results. This is consistent with the findings of Yang et al. [9], who reported positive results in 58.5% of infiltrative lesions [9]. However, we did not identify a statistically significant association between the morphological features of endoscopic lesions and LBC results.

There are several limitations to our study. First, the population of patients diagnosed with disorders other than lung cancer was small, with only 14 individuals. This could have confined the detection of false positives in the LBC analysis. Second, it was not feasible to conduct follow-up examinations until the complete disappearance of the lesion in patients diagnosed with conditions other than cancer, thus precluding the possibility of ruling out cancer. Third, having CS interpreted by a technician and LBC interpreted by a pathologist may lead to interobserver variability. However, we have required that the technician and pathologist have more than five years of experience and the same analyst throughout the study, thus minimizing the potential for reading errors. Finally, the study exclusively used cytologic fluid materials, and the gold standard for diagnosis was established based on the histopathological examination of tissue biopsies. This investigation did not explore the approach of acquiring a cell block of BAL in conjunction with LBC. It is assumed that the concurrent use of cell block and LBC of BAL can enhance diagnostic accuracy, sensitivity, and specificity for BAL sampling, particularly when complemented with immunohistochemical stains [24,25].

## Conclusions

The application of LBC for BAL fluid has shown the possibility of heightened diagnostic effectiveness relative to CS for diagnosing lung cancer. We suggest using a higher volume of fluid for LBC to enhance the sensitivity of lung cancer diagnosis. Further studies are needed to confirm this hypothesis.
